# Can the Supplementation of Oocytes with Extra Copies of mtDNA Impact Development Without Being Transmitted? A Molecular Account

**DOI:** 10.3390/ijms26062746

**Published:** 2025-03-18

**Authors:** Justin C. St. John, Eryk Andreas, Alexander Penn

**Affiliations:** Experimental Mitochondrial Genetics Group, School of Biomedicine, Faculty of Health and Medical Sciences, The University of Adelaide, Adelaide Health and Medical Sciences Building, Adelaide, SA 5000, Australia; eryk.andreas@adelaide.edu.au (E.A.); alexander.penn@adelaide.edu.au (A.P.)

**Keywords:** mtDNA, supplementation, transmission, segregation, oocyte, gene expression, DNA methylation, offspring

## Abstract

The introduction of extra copies of mitochondrial DNA (mtDNA), whether autologous or heterologous, into oocytes at the time of fertilisation or through other assisted reproductive technologies, such as nuclear transfer, is a contentious issue. The primary focus has been on whether third-party mtDNA is transmitted to the offspring and if it impacts offspring health and well-being. However, little attention has focused on whether the introduction of extra copies of mtDNA will interfere with the balance established between the nuclear and mitochondrial genomes during oogenesis and as the developing embryo establishes its own epigenetic imprint that will influence mature offspring. Whilst we determined that sexually mature offspring generated through mtDNA supplementation did not inherit any-third party mtDNA, they exhibited differences in gene expression from three tissues derived from three separate embryonic lineages. This resulted in a number of pathways being affected. In each case, the differences were greater in the heterologous and autologous comparison than when comparing all supplemented offspring against non-supplemented offspring. Many of the changes in gene expression were coupled to differential DNA methylation across tissues, some of which were tissue-specific, with high levels observed in the heterologous against autologous comparison. An analysis of DNA methylation in blastocyst-stage embryos pointed to changes in patterns of DNA methylation that were transmitted through to the offspring. Our results indicated that extra copies of mtDNA may not be transmitted if introduced at low levels, but the changes induced by supplementation that occur in DNA methylation and gene expression in the blastocyst have a profound effect on tissues.

## 1. Introduction

A number of the more invasive and sophisticated reproductive technologies have the potential for two populations of mtDNA (heteroplasmy) to persist in the reconstructed oocyte and, thus, be transmitted through the embryo to the fetus and, ultimately, the offspring [1]. These technologies include, for example, the various forms of nuclear transfer and mitochondrial supplementation. In the case of nuclear transfer, the donor nucleus is either removed from one oocyte or zygote and introduced into an enucleated recipient oocyte or zygote from the same stage of development [2,3], or a somatic or embryonic cell is introduced into an enucleated oocyte [4]. With mitochondrial supplementation, purified populations of mitochondria are directly introduced into the oocyte at the time of fertilisation [5,6]. In some cases, an autologous (same genetic source) approach is followed, whilst, in others, the application is heterologous (third-party) [6,7].

mtDNA is located within each mitochondrion and is approximately 16.6kb in size, circular, and double-stranded, comprising a heavy and light strand [8]. It is critical for high-OXPHOS-requiring cells and tissues, as it encodes key genes of the electron transfer chain [9]. In all, it encodes seven subunits of Complex I, one of Complex III, three of Complex IV, and two of Complex V [8]. It also encodes 2 *rRNA*s and 22 *tRNA*s. Rearrangements (mutations, deletions, and insertions) of these genes can lead to an increasing number of diseases, some of which are severe, if not fatal [10]. mtDNA also has two non-coding regions, with the larger, namely the displacement loop, housing two hypervariable regions that are frequently used to identify maternal relatives and regulatory regions that interact with the nuclear-encoded mtDNA-specific replication factors that first drive the transcription and then replication of this genome [11]. The other smaller region houses the origin of replication for the light strand. mtDNA is primarily only maternally inherited and, for each individual [12], specifically from the population of mtDNA present in its originating oocyte at the time of fertilisation.

The addition of extra copies of mtDNA through nuclear transfer, ooplasmic transfer, and mitochondrial supplementation has the potential for offspring to inherit two populations of mtDNA when third-party mtDNA is used [1]. The mode of selection for third-party mtDNA is yet to be fully understood. However, it is evident that the introduction of a nucleus with either mitochondria attached or extra mitochondria alone into an oocyte drives an mtDNA replication event at the two-cell stage, which was previously deemed to be a turnover checking mechanism, but can result in a full-blown mtDNA replication event [5]. This turnover event is normally the only mtDNA replication event that takes place during preimplantation development until the blastocyst stage, when mtDNA replication is restricted to the trophectodermal cells that give rise to the placenta [13]. The cells of the inner cell mass that give rise to embryonic stem cells [14], which are the precursors of the embryo proper and fetus [15], do not replicate mtDNA until post gastrulation [16,17]. However, embryos derived through nuclear transfer continue to replicate their mtDNA throughout preimplantation development, and, thus, the small amount of mtDNA carried over can be selected for or against [18]. In cases where it is selected, it can account for up to 44% of the total mtDNA content in some porcine tissues [19] and up to 60% in the body fluids and peripheral tissues of a human at birth [20].

In previous work, we generated porcine founders and a subsequent generation through mtDNA supplementation. We conducted our studies in pigs, since they are the most appropriate model to study human development. Many of the pig’s organ systems and physiological and pathophysiological responses are similar to those of the human [21], as are its embryology, early development [22], and mtDNA copy number, replication, and reduction events during development [13]. In all, by introducing an extra ~780 copies of mtDNA into metaphase II oocytes, we generated seven autologous and five heterologous founders [6]. We assessed the progeny from birth through to sexual maturity for development and growth rates and observed significant differences compared to our control population. Furthermore, we observed a number of differences for biochemical and haematological indicators at weaning, some of which normalised at sexual maturity. Nevertheless, when we assessed high-OXPHOS-requiring tissues, we identified a number of genes that were differentially expressed and affected some key pathways and networks. However, the female offspring were fertile, as they produced litters consistent in size with Australian commercial pig breeding lines. We also conducted mtDNA sequencing analysis, primarily on tail samples, to confirm whether autologous supplementation had successfully taken place.

Since we observed a number of significant differences in the supplemented offspring, we conducted a more comprehensive analysis at a genomic level. We first sought to determine if the mtDNA integrity of the offspring and their progeny was compromised by the transmission of third-party (heterologous) mtDNA or the mtDNA from sister oocytes (autologous). We undertook this by including all tissues from six founders and cohorts of oocytes from some females. We then determined the degree of differential gene expression in all supplemented pigs compared to controls and between heterologous- and autologous-derived pigs by performing RNA-seq on three tissues from six of the supplemented animals. We then sought to determine if the differences in gene expression arose through differential DNA methylation using reduced representation bisulfite sequencing (RRBS). Finally, we sought to determine whether the differential DNA methylation and gene expression arose from changes in DNA methylation and gene expression in blastocysts generated through mtDNA supplementation. We report that the offspring maintained mtDNA genomic integrity irrespective of whether they received autologous or heterologous mtDNA supplementation. However, there were differences in gene expression and DNA methylation in the tissues of these animals when compared with controls and greater differences between the autologous and heterologous cohorts. We further identified specific DNA methylation traits in blastocyst-stage embryos that were transmitted to offspring.

## 2. Results

### 2.1. Assessment of the mtDNA Integrity of Autologous Offspring

**Figure 1:**
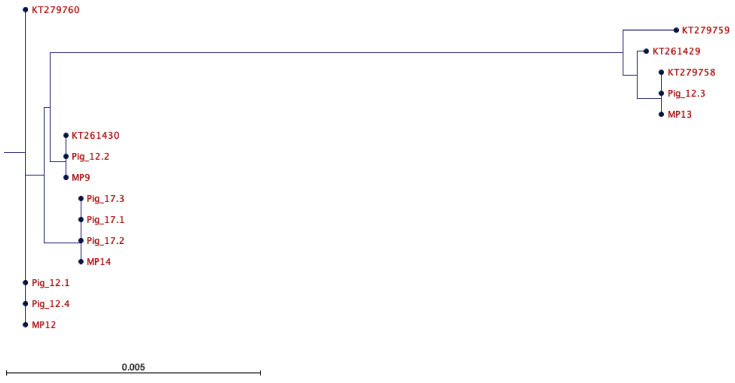
Phylogenetic representation of autologous offspring. Phylogenetic tree for the offspring derived from autologous mtDNA supplementation (12.1, 12.2, 12.3, 12.4, 17.1, 17.2, and 17.3) and the respective mtDNA isolate (MP9, MP12, MP13, and MP14) used in their generation. Known mtDNA haplotypes are KT279758, KT261429, KT279759, KT261430, and KT279760, showing the relationship of the autologous derived pigs with mtDNA haplotypes present in commercial breeding populations. The scale bar is indicative of genetic (evolutionary) change. Evolutionary change runs horizontally and samples with the same mtDNA sequence will align vertically. In this instance, ‘0.005’ represents 0.005 genetic change determined by nucleotide substitutions (mutations, insertions, and deletions) at each site within the genome, i.e., the number of changes or ‘substitutions’ as a function of sequence length. There are 15 substitutions between KT279758 and KT261429; 32 substitutions between KT261429 and KT279759; 202 substitutions between KT279759 and KT261430; and 17 substitutions between KT261430 and KT279760.

We generated seven offspring from two rounds of autologous mtDNA supplementation. Figure 1 and Table 1 show the relationship of the offspring with their respective mitochondrial preparation (MP) that was used for the supplementation process and their relationship with previously identified mtDNA haplotypes within Australian commercial pigs. In all, we analysed a tail sample from Pig 12.1; tail and oocytes from Pigs 12.2, 12.3, 17.1, and 17.3; and a full range of tissues from Pigs 12.4 and 17.2. Each of the offspring harboured several insertions and deletions which appeared to be ubiquitously shared among all offspring and, therefore, did not appear to represent genetic rearrangements resulting from the mtDNA supplementation approach (Supplementary Table S1). Furthermore, we did not observe the transmission of any unexpected heteroplasmy to the founders’ tissues or oocytes that would give rise to the next generation. Consequently, there was no discernible transmission of mtDNA that would question the mtDNA genetic integrity of the offspring.

### 2.2. Assessment of the mtDNA Integrity of the Heterologous Cohort

**Figure 2:**
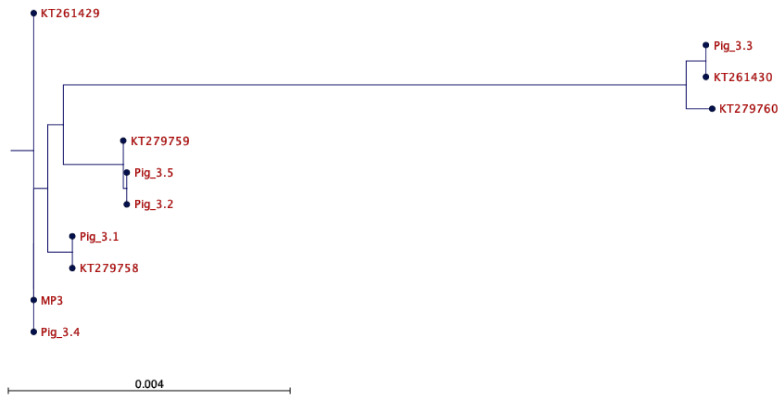
Phylogenetic representation of heterologous offspring. Phylogenetic tree representing the offspring derived from heterologous mtDNA supplementation (3.1, 3.2, 3.3, 3.4, and 3.5) and the mtDNA isolate (MP3) used in their generation. The offspring are contrasted with known mtDNA haplotypes KT279758, KT261429, KT279759, KT261430, and KT279760 to demonstrate their relationship to commercial breeding populations. The scale bar, ‘0.004’, represents 0.004 genetic change determined by nucleotide substitutions (mutations, insertions, and deletions). The substitution rates are the same as those for Figure 1.

For the heterologous cohort, there were again several insertions and deletions present, but within the same range as that for the autologous cohort. Figure 2 and Table 1 show the relationship between the founders, the mitochondrial preparation used to supplement the oocytes (MP3), and recognised mtDNA haplotypes in Australian commercial pigs. Nevertheless, MP3 was not present in any of the offspring, either in tail samples only (Pig 3.5) or all tissues (Pigs 3.1 to 3.4) (Supplementary Table S2). Furthermore, the assessment of oocytes from the female founder (Pig 3.3) also highlighted that the supplement was not present in her oocytes, any of the tail samples of her progeny that had been generated through natural conception (Pigs 3.3.1 to 14), or the oocytes of 3.3.14 (Supplementary Table S2). Interestingly, Pig 3.4 possessed the same mtDNA genotype as MP3 and was, therefore, reclassified as autologous.

### 2.3. Assessment of Mitochondrial Preparations for Genetic Integrity

Given that the mitochondrial preparations were derived from oocytes that had failed to reach the metaphase II stage of development following in vitro maturation, we assessed the samples used for the supplementation process along with others to determine if they carried variants which, if transmitted, could potentially pose a risk for any offspring generated using this approach. In each case, we did not observe any variants present at greater than 7.3% in the MP samples (Supplementary Table S3), except at nt13748 in MP12, which was transmitted at a lower rate in the tissues of Pig 12.4 and is highly likely to be indicative of natural heteroplasmy present in the female germline given its high load in the MP sample. Since ~780 copies of mtDNA were introduced into the oocyte at the time of fertilisation and the variant rate was 7.3%, this equates to 57 copies that would carry a variant. As a contribution to the fertilised oocyte, which contains circa 323,000 copies of mtDNA [13], copies carrying the highest level of variant represented 0.018% of the fertilised oocyte’s mtDNA content. This would require a considerable replicative advantage to induce a phenotypic effect.

### 2.4. Gene Expression for All Supplemented Offspring

**Figure 3:**
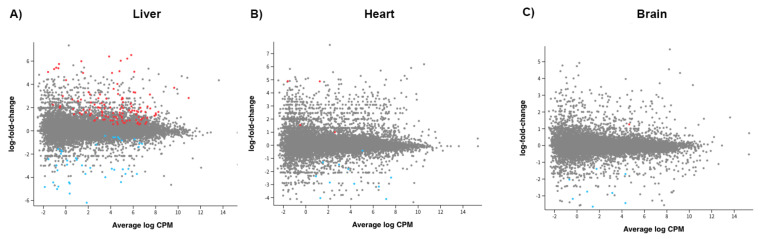
Graphical representation of the distribution of genes expressed in liver (**A**), heart (**B**), and brain (**C**) tissue for the comparison between all offspring derived through mtDNA supplementation and controls. A statistical cut off point of FDR <0.05 was employed. Red indicates genes significantly upregulated and blue downregulated. CPM = counts per million.

To determine if additional mtDNA altered the gene expression profiles of the adult offspring, we performed RNA-seq on three sets of tissues, namely the liver, brain, and heart, which primarily rely on OXPHOS for the generation of ATP, have a high mtDNA copy number, and are susceptible to harbouring mtDNA rearrangements. We mapped the sequences to the latest pig assembly. Firstly, we compared all offspring that had been generated through mtDNA supplementation (n = 6) against naturally conceived controls (n = 3), since these are the most appropriate control group for assessing the effects of assisted reproductive technologies on the wellbeing of human offspring [23,24,25,26]. In the liver, there were 161 differentially expressed genes (DEGs; False Discovery Rate—FDR < 0.05; Figure 3A; Supplementary Table S4), whilst in the heart (Figure 3B; Supplementary Table S4), there were 15, and in the brain, 9 (Figure 3C; Supplementary Table S4). Across the three tissues, LOC110260659 (a translationally controlled tumour protein pseudogene) and LOC100518848 (40S ribosomal protein S21) were common to all three, whilst LOC110258138 (non-coding RNA) and *SGCA* were common to the brain and liver and *PDZD9* and LOC106505031 (non-coding RNA) were common to the heart and liver (Table 2; Supplementary Table S4).

We then expanded the cut-off criterion for the data sets to *p* < 0.05 and conducted gene ontology (GO) analyses using edgeR. Expanding the data sets in this manner allowed for a more comprehensive analysis and discounted false negatives from exclusion, as shown in [27]. The GO terms for each tissue are listed in Supplementary Table S5. The top three for each tissue were the extracellular region, cell fate determination, and extracellular space for the brain; cellular amino acid metabolic process, cellular amino acid catabolic process, and channel activity for the heart; and gated channel activity, potassium ion transmembrane transporter activity, and potassium channel activity for the liver. There were several GO terms that were common between two tissues but not common amongst the three tissues (Supplementary Table S5).

**Figure 4:**
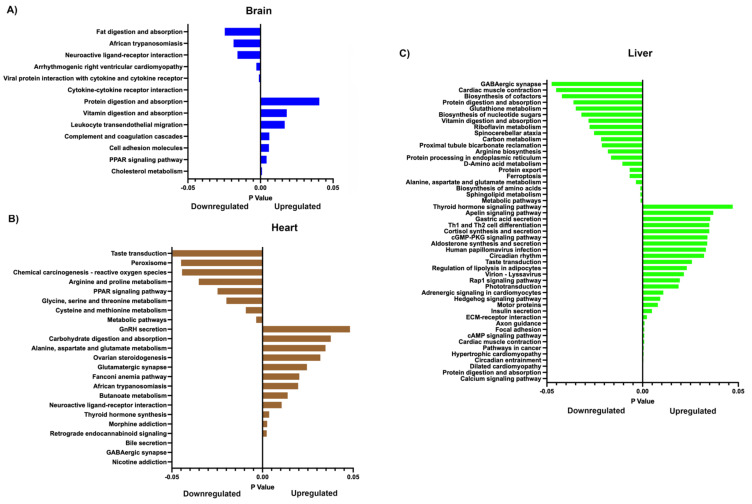
Graphical representation of significantly different down and upregulated pathways for brain (**A**), heart (**B**), and liver (**C**), as determined by KEGG, for all offspring when compared against controls. Unadjusted *p* < 0.05. The full list of pathways is presented in Supplementary Table S5E–G.

When applying pathway analysis using Kyoto Encyclopedia of Genes and Genomes (KEGG) and assessing by downregulation only, as demonstrated in [27,28], the top three downregulated pathways were cholesterol metabolism, the PPAR signalling pathway, and cell adhesion molecules in the brain (Figure 4A); metabolic pathways, cysteine and methionine metabolism, and glycine, serine and threonine metabolism in the heart (Figure 4B); and metabolic pathways, sphingolipid metabolism, and the biosynthesis of amino acids in the liver (Figure 4C). The top three upregulated pathways were cytokine–cytokine receptor interaction, viral protein interaction with cytokine and cytokine receptors, and arrhythmogenic right ventricular cardiomyopathy for the brain (Figure 4A); nicotine addiction, GABAergic synapse, and bile secretion for the heart (Figure 4B); and the calcium signalling pathway, protein digestion and absorption, and dilated cardiomyopathy for the liver (Figure 4C). Overall, a number of metabolic, signalling, and infection pathways were altered.

### 2.5. Gene Expression Based on Source of mtDNA Supplementation

**Figure 5:**
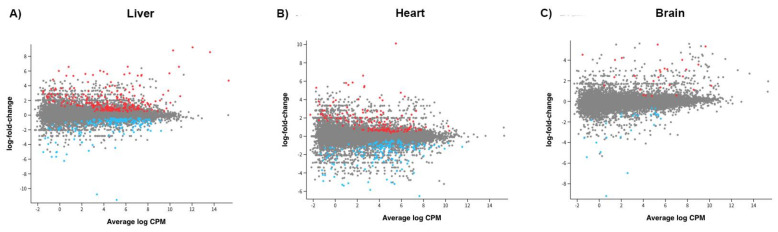
Graphical representation of the distribution of genes expressed in liver (**A**), heart (**B**), and brain (**C**) tissue for the comparison between heterologous and autologous offspring derived through mtDNA supplementation. A statistical cut-off point of FDR < 0.05 was employed. Red indicates genes significantly upregulated and blue downregulated. CPM = counts per million.

We then assessed the effects of the source of the mtDNA used in the process of supplementation on tissue gene expression. In this instance, there were 392 significant DEGs for the liver (Figure 5A); 312 for the heart (Figure 5B); and 52 for the brain (Figure 5C) based on the comparison between autologous and heterologous supplementation (Supplementary Table S6). In all, three genes were common to the three tissues, LOC102164558, LOC100525311, and LOC100152036, and 19 were common to two tissues, *ACSM5*, *IGF2BP3*, *KIRREL*, LOC106508088, *TLE4*, *TRIM7*, *NTRK2*, LOC106509396, *COG7*, LOC110261018, *EMILIN3*, LOC106506003, *RRBP1*, LOC102163231, *FLRT3*, LOC110258845, LOC106506130, *NEXN*, and *SPAG16* (FDR < 0.05; Table 3; Supplementary Table S6). Notably, these genes were associated with transcription, immunoglobin receptivity, Golgi, and metabolism.

**Figure 6:**
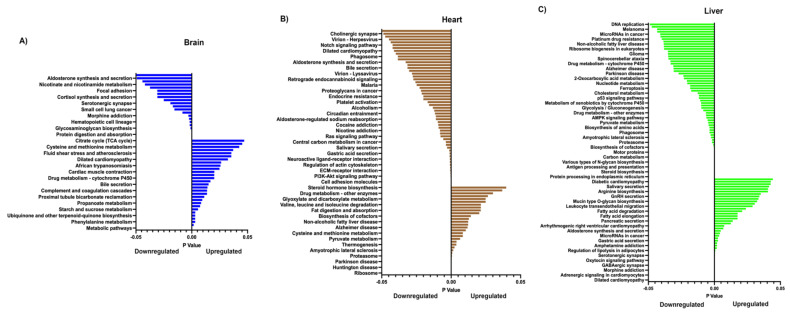
Graphical representation of significantly different down and upregulated pathways for brain (**A**), heart (**B**), and liver (**C**), as determined by KEGG, for the comparison between heterologous against autologous offspring. Unadjusted *p* < 0.05. The full list of genes differentially up and down regulated is presented in Supplementary Table S7E–G.

The expanded gene list revealed a large number of GO terms that were affected (Supplementary Table S7). For the brain, in terms of cellular components, the top three were extracellular region, extracellular space, and cell periphery. For molecular function, the affected terms were endopeptidase inhibitor activity, peptidase inhibitor activity, and molecular transducer activity. The affected biological processes were the positive regulation of the cytosolic calcium ion concentration, system process, and the phospholipase C-activating G protein-coupled receptor signalling pathway. The affected top three upregulated pathways for the brain were metabolic pathways, the PPAR signalling pathway, and Phenylalanine metabolism, whilst the downregulated pathways were neuroactive ligand–receptor interaction, protein digestion and absorption, and ECM–receptor interaction (Figure 6A; Supplementary Table S7).

For the heart, the top three GO terms for cellular components were cytosolic ribosomes, ribosomes, and ribosomal subunits; for molecular function, they were the structural constituents of ribosomes, structural molecule activity, and extracellular matrix structural constituents; and for biological processes, they were the peptide biosynthetic process, translation, and the peptide metabolic process (Supplementary Table S7). The affected top three upregulated pathways were ribosomes, coronavirus disease (COVID-19), and Huntington’s disease, with OXPHOS ranked fourth (Figure 6B; Supplementary Table S7). The top downregulated pathways were protein digestion and absorption, cell adhesion molecules, and GABAergic synapse.

For the liver, the top three GO terms for cellular components were polymeric cytoskeletal fibres, supramolecular fibres, and supramolecular polymers; for molecular function, they were ligand-gated channel activity, ligand-gated ion channel activity, and intracellular ligand-gated ion channel activity (Supplementary Table S7). The top upregulated pathways were dilated cardiomyopathy, hypertrophic cardiomyopathy, and adrenergic signalling in cardiomyocytes, whilst the top downregulated pathways were protein processing in the endoplasmic reticulum, metabolic pathways, and steroid biosynthesis (Figure 6C; Supplementary Table S7). We further note that, across the three tissues, a number of pathways associated with metabolism, including the tricarboxylic acid (TCA) cycle, infection, cell cycle, and steroidogenesis, were affected. Overall, key pathways associated with metabolism, signalling, and steroidogenesis appeared to be affected.

### 2.6. Reactome Pathway Analysis

We further undertook Reactome pathway analysis to determine if the differences in gene expression resembled pathways associated with disease and reported altered pathways. For all offspring compared with controls, 174 pathways were deemed to be affected through upregulation or downregulation for the brain. Likewise, for the heart and liver, 98 and 149 pathways, respectively, were significantly affected (Supplementary Table S8). The top 10 upregulated and downregulated pathways are shown in Table 4. A number of pathways were common to two tissues in both the upregulated and downregulated cohorts, whilst CDK-mediated phosphorylation and the removal of CDC6 were common to all three tissues in the downregulated cohort (Supplementary Table S8). In terms of the autologous and heterologous cohorts, 130 pathways were differentially regulated in the brain, 106 in the heart, and 169 in the liver (Table 5; Supplementary Table S9). Furthermore, there were a number of pathways that were upregulated or downregulated in a similar manner between two of the tissues, but none were common to all three tissues (Supplementary Table S9). Again, overall, a number of pathways associated with cell cycle, metabolism, and, in this instance, the inflammasome were affected for all supplemented offspring and the heterologous against autologous cohorts.

### 2.7. DNA Methylation Analysis

We undertook reduced representation bisulfite sequencing to determine if the process of supplementation induced changes in the DNA methylomes of the supplemented-derived offspring (see Supplementary Table S10A–C). Employing an FDR of < 0.05, there were 203 identifiable differentially methylated sites which were present in coding and non-coding regions in brain tissue. A number of these regions were differentially methylated at multiple sites, as highlighted in Supplementary Table S10. Similarly, there were 161 sites in the heart and 362 sites in the liver. Across the tissues, there was overlap amongst differentially methylated sites, as indicated in Supplementary Table S10D. As with the comparison of gene expression between autologous and heterologous pigs, we saw increased numbers of methylated CpG sites (Supplementary Table S10E–G). The brain exhibited 1026 differentially methylated sites, many of which populated a coding or non-coding region more than once. Likewise, there were 637 sites in the heart and 1486 sites in the liver, which frequently appeared multiple times in associated regions. A number of sites were also common to two or more tissues and between the comparisons of all offspring against controls and the heterologous against the autologous cohorts (Supplementary Table S10H); and between all offspring and the heterologous against the autologous cohort for each tissue (Supplementary Table S10I–K).

### 2.8. Overlap of Differentially DNA Methylated Regions and Differentially Expressed Genes

To determine if differential DNA methylation overlapped with differential gene expression, we firstly assessed all offspring (Table 6; Supplementary Table S11A–C). In this case, differential methylation overlapped with gene expression for *ADRM1* and *RPS21* (brain), *CHRNA4* and *MYT* (heart), and *PTK6*, *KIF1A*, *ABCA13*, *NOS2*, *DSCAM*, *NAV3*, and *SDK1* (liver). The number of affected genes increased in the heterologous against autologous comparisons (Table 6; Supplementary Table S11D–F). There were 9 genes affected in the heart and 19 in the liver, whilst in the brain, the number of affected genes remained at 2. Except for *ACSM5* in the heterologous against autologous comparisons for the brain and heart, none of the other affected genes appeared in more than one tissue (Table 6; Supplementary Table S11G). Indeed, the differential profiles were very distinct for each tissue type and the method of supplementation.

### 2.9. Origin of the Differential DNA Methylation and Gene Expression

Since we observed a number of differentially methylated regions that were common to two or all three tissues, we investigated whether they had a common origin. We chose to examine expanded blastocysts, a stage of development where the resetting of the DNA methylome is anticipated to be complete or nearing completion [29]. In this instance, we returned to previous data sets that had identified differentially methylated regions (DMRs) in expanded blastocysts generated through autologous mtDNA supplementation [30]. Of the 2199 differentially methylated regions, we were able to identify 1176 annotated DMRs (Supplementary Table S12). Of these 30 (brain), 24 (heart), and 58 (liver) blastocyst DMRs overlapped with the differentially methylated sites in their respective tissues when all offspring were compared with controls, 8 of which were common to all three tissues and 14 to two tissues (Table 7). For the heterologous against autologous cohort comparison, 105 (brain), 78 (heart), and 175 (liver) DMRs overlapped with differentially methylated sites in the tissues (Supplementary Table S12), with 43 sites common to all tissues and 53 to two tissues (Table 7). Between all offspring and the heterologous against autologous cohorts, only two regions, *PITRM1* and *PTPRN2*, were common to all three tissue types, whilst none were common to two tissue types. Finally, we analysed the overlap in gene expression, DNA methylation, and blastocyst DMRs for each cohort. For the cohort of all offspring, only *ABCA13*, *NAV3*, and SDK1 in the liver were present as DMRs in the blastocysts, whilst for the autologous and heterologous cohorts, ACSM5 (brain and heart), *DGKI*, *MYLCD* (heart), *DLGAP2*, *NAV2*, *ATP8A2*, *LRRC8D*, *SMURF1*, *PPFIBP2*, and *CFAP46* (liver) were differentially methylated in blastocysts. These genes are related to metabolism, axonal direction and migration, and inflammation. In all, this highlights the degree of diversity of the differential methylation present in expanded blastocysts following supplementation and tissue-specific segregation (Supplementary Table S12).

## 3. Discussion

We developed a large animal model to specifically ask whether altering the balance between the nuclear and mitochondrial genomes in the oocyte would affect nuclear gene expression and DNA methylation in resultant offspring. Previously, we showed that there are differences at the physiological, biochemical, and anatomical levels [6]. Here, we examined the changes at a molecular level. Although our extensive analyses of tissues and oocytes revealed that no third-party mtDNA was transmitted to resultant offspring, nor was mtDNA integrity affected following both heterologous and autologous mtDNA supplementation, there were significant changes in nuclear gene expression between the offspring generated through mtDNA supplementation and those that were not. Furthermore, there were greater distinctions when the animals were assessed based on whether they had received mtDNA from autologous or heterologous sources.

The amount of mtDNA introduced into an oocyte was minimal, but, as previously shown, sufficient to rescue oocytes deficient in mtDNA [5]. It is likely that the introduction of the extra copies of mtDNA was recognised by the fertilised oocytes, since our previous work showed that the introduction of similar amounts of mtDNA into mtDNA deficient oocytes triggered an mtDNA replication event [5]. This, in turn, also resulted in significant changes in gene expression by the blastocyst stage of development [5]. Indeed, this mtDNA replication event arises from an mtDNA turnover event that takes place between fertilisation and the two-cell stage event [13], most likely similar to a cell cycle checkpoint event with minimal increases in copy number. Nevertheless, in mtDNA-deficient oocytes (<50,000 copies of mtDNA), the process restores the mtDNA copy number to pre-fertilisation levels indicative of fertilisable oocytes (>200,000) [5] and is mediated by changes in the DNA methylation of the mtDNA-specific replication factor POLG [31]. Given that an mtDNA replication takes place, it is highly unlikely that the postulated restoration in embryo development [32,33] arises from adding extra units of energy into an oocyte to mediate the rescue process. Indeed, an additional 780 copies of mtDNA would provide between 390 and 780 extra mitochondria to an oocyte that already has circa 50,000 to 100,000 mitochondria present (based on oocyte mitochondria possessing between one and two copies of mtDNA each [34]). Indeed, during preimplantation development, the embryo normally reduces its mtDNA content [35] as each newly formed cell divides and, since there is no further mtDNA replication in embryonic cells until post-gastrulation [16,17], the need for OXPHOS-mediated energy production is limited, as the greater dependency is for glycolysis [36]. Consequently, preimplantation development is programmed to actively reduce mtDNA copy number whilst the process of mtDNA supplementation mediates a genomic event rather than a metabolic event. From our current work, this extends to a series of epigenetic events. Interestingly, the supplement contained very low levels of rearrangement, which is more indicative of the level of common deletion observed in oocytes (>0.1%) [37] than the highly overestimated levels of common deletion incorrectly reported by others [38].

Although a limited number of genes were differentially expressed in the three tissues analysed at FDR <0.05, the expansion of the gene sets to conduct downstream analyses showed that a significant number of GO terms and key pathways were affected. The affected pathways tended to be distinct to each tissue type, primarily because of the limited overlap between genes that were differentially expressed in each tissue type. However, amongst the downregulated cohort, there was commonality to the extent that various metabolic pathways were affected, though many of these were not related to the mitochondrial-genome-encoded genes. Nevertheless, many were associated with amino acid and carbon metabolism, which suggests implications for DNA replication and gene regulation. Amongst the upregulated pathways, there was a bias toward cytokine regulation (brain) and cardiomyopathy. Indeed, in mouse studies of mtDNA supplementation, we observed a severe form of heart defect that was transgenerationally transmitted [39].

Our outcomes show that the differences between autologous and heterologous supplementation appear to have a greater effect on gene expression and DNA methylation than both sets of supplemented offspring combined compared to non-supplemented controls. Indeed, this suggests that third-party mtDNA induces a differential response, as observed through cytokine responses and the activation of inflammasome pathways. It is evident that mtDNA can interact with various immune factors to trigger a response and, as the mitochondrial genetic code is slightly different from the nuclear genome coupled with the sequence differences associated with the exogenous source of mtDNA, it is likely that third-party mtDNA would be considered as ‘foreign’ [40,41]. This, in turn, could contribute to autoimmune-like syndromes [42,43], as is the case with the by-directional leakage of maternal and fetal DNA during pregnancy, which leads to microchimerism [44]. This is further supported by the necessity for the active degradation of paternal mtDNA shortly after fertilisation through mitophagy [45]. Indeed, in larger mammals, paternal mtDNA only seems to persist in interspecific crosses such as Indian and Chinese rhesus macaque [46] and *Bos indicus* and *taurus* crosses [47].

Whilst there were some commonly differentially expressed genes, there were also a number of very distinct tissue-specific differential gene expression profiles associated with mtDNA supplementation (all offspring and autologous vs heterologous), where the brain exhibited the fewest differences and the liver the most. This was also the case for the respective DNA methylation profiles. Each of the tissues we analysed arose from different embryological origins [48]. The brain primarily arises from the ectoderm, the heart from the mesoderm, and the liver the endoderm, although this is not entirely exclusive. For example, >70% of cells in the liver (hepatocytes and cholangiocytes) arise from the endoderm, whilst hematopoietic, Kuppfer, stromal, and stellate cells originate from the mesoderm [49]. Nevertheless, it is feasible that each of the germ layers was differentially affected by mtDNA supplementation during early development, which would account for the skewed segregation of DNA methylation marks and their effects on tissue-specific gene expression. However, the question that then remains to be answered is why such a bias takes place, given that all cells in the early preimplantation embryo start from a single fertilised cell that then rapidly divides, otherwise regarded as a form of clonal expansion. Perhaps the processes induced by supplementation are partial or incomplete processes that, during preimplantation development, result in some of the dividing genomes being fully or partially de/methylated. Previous results inform us that different mtDNA haplotypes have different impacts on the same nuclear genotype, where alterations in the TCA cycle result in metabolite activity that modulates the TET family of proteins, which, in turn, act on DNA methylation [50]. If this activity is sporadic across the developing embryo, in which TET proteins play a major role [51], then the effects on specific lineages could, indeed, be random but tissue-bias-specific. This would, thus, suggest that another effect is in play. In this instance, if a number of metabolic factors are at play as a result of supplementation in the developing embryo, the cells harbouring the affected genomes may be skewed to the endodermal lineages and the liver specifically, given its high metabolic profile as a tissue. This would be similar to environmental factors or embryo culture media influencing DNA methylation [52], and the effects on methylation would be marked.

Whilst our gross anatomical, physiological, and biochemical analyses suggested that mild to moderate effects arose from mtDNA supplementation [6], more recently, we reported changes in the metabolite profiles of supplemented pigs at sexual maturity, which could impact their health and well-being. If we compare gene expression (Figure 6; Supplementary Table S7E–G) and our previous metabolite profile analysis [53] conducted on the same tissues of the same animals, we find that several affected KEGG metabolism pathways exhibited similar patterns. These include glyoxylate and dicarboxylate metabolism and pyruvate metabolism, which were downregulated in the liver tissue from heterologous-derived pigs. However, some others had opposite patterns. For instance, we found that starch metabolism in the brain and glycine, serine, and threonine metabolism and glyoxylate and dicarboxylate metabolism in heart tissue derived from the heterologous cohort were upregulated based on gene expression analysis, but were downregulated based on metabolite profile analysis. Nevertheless, this phenomenon can be explained by the actions of mRNA post-transcriptional modification processes, including RNA splicing, ubiquitination, and degradation. Therefore, gene expression is not always positively correlated with its protein expression [54], and the concordant and discordant expression between the gene and its protein can be varied in a tissue-specific manner [55].

Although we used data sets derived in different ways to assess the longitudinal impact on tissue DNA methylation and gene expression, it is evident that the DNA methylome is set during very early development and that characteristic changes impact offspring. Indeed, in mice, we observed changes in gene expression in the primordial follicles of offspring derived from supplementation [39]. Whilst the fertilising sperm contributes to the newly derived methylome of the developing embryo [56], altering its mtDNA content, whether by autologous or heterologous supplementation, this adds a degree of complexity that could have implications for offspring health and well-being. Our longitudinal analysis shows that the epigenetic processes established during oogenesis and spermatogenesis and reestablished following fertilisation, once recombination has taken place, can be modified as a result of supplementation. Given that supplementation takes place prior to recombination and that the extra mtDNA induces epigenetic modifications, it is apparent that the process of DNA methylation preserves these newly induced imprints, with modified gene expression present at the blastocyst stage and the retention of some modifications later during development, as seen in sexually mature offspring. Although the imprinted genes do not appear to be grossly affected [57], there is a degree of commonality with somatic cell nuclear transfer, which is dependent on the recipient oocyte’s cytoplasm to promote the reprogramming of the somatic nucleus and forge an embryonic methylome [58]. Indeed, this assisted reproductive technology is confounded by the presence of third-party mtDNA [18,19]. Perhaps some of the failure to completely reprogramme the somatic nucleus is due the ‘mtDNA effect’, especially as the third-party source of mtDNA can contribute to the offspring’s mtDNA genotype in a tissue-specific manner [19]. In a bovine model of somatic cell nuclear transfer, where the donor the cells, derived from a specific primary cell line, were depleted of their mtDNA, blastocysts exhibited 509 differentially expressed genes as a result of adding 563.1 ± 57.7 (mean ± SEM) extra copies of mtDNA to the oocyte as somatic cell nuclear transfer was performed [59].

As with any new assisted reproductive technology, its degree of societal and scientific tolerance needs to be established before it is safe to introduce it. Importantly, simply reporting a successful live birth is not just the answer, but strict follow-up studies need to be conducted that assess all developmental milestones and monitor for aberrant mtDNA transmission. Our results point to anomalies that could have implications for offspring health and well-being and should be duly noted and reflected upon when furthering this form of treatment clinically. Consequently, those having practiced mitochondrial supplementation or similar approaches clinically [7,60,61] should consider the long-term effects of this approach and ensure that full-scale follow-up studies are implemented. Furthermore, our supplementation process acts as a model for mtDNA carryover (a third-party source) for mitochondrial donation, a form of nuclear transfer technology designed to alleviate the effects of mtDNA diseases [62,63] or failed fertilisation outcomes [20] without interference from the third-party mtDNA when autologous supplementation is applied. Our results suggest that mtDNA carryover, which normally results in a greater contribution of third-party mtDNA (circa 2000 copies of mtDNA cf. 780 copies), would alone induce epigenetic changes that would compound the effects of the nuclear transfer itself. In addition, others seek to add agents such as the anti-oxidant MitoQ and resveratrol to restore mitochondrial dysfunction and resultantly increase the mtDNA copy number in oocytes [64,65], which, in turn, will affect the balance between the two genomes and affect the developing methylome in a longitudinal manner.

In conclusion, in a large animal model that is highly relevant to human development, it is evident that mtDNA supplementation can result in the generation of sexually mature offspring that do not carry third-party mtDNA, and the source of mtDNA does not affect the animals’ mtDNA genetic integrity. However, the source of mtDNA can impact gene expression through altered patterns of DNA methylation that are initiated very early during development and impact the DNA methylation status of blastocysts. Some of these imprints are then transmitted through to the offspring, some to more than one tissue and others in a tissue-specific manner. This transmission is either indicative of targeting to specific DNA molecules during early development or a form of randomised methylation that influences or partially influences tissue-specific gene expression. Nevertheless, an array of pathways associated with metabolism, cell cycle regulation, gene regulation, inflammation, and infection are affected.

## 4. Materials and Methods

### 4.1. Animal Ethics

All procedures that involved the generation and use of live animals were assessed by the University of Adelaide Animal Ethics Committee and approved under number 32293 (original approval date: 29 March 2018). Piglets and recipient gilts were housed in a temperature-controlled room. Piglets received a vaccination and were allowed access ad libitum to a commercial standard diet and water, as described in [6].

Unless otherwise stated, the chemicals used in this project were supplied by Merck Life Science Pty Ltd. (Bayswater, VIC, Australia).

### 4.2. Collection of Sus Scrofa Cumulus–Oocyte Complexes and In Vitro Maturation

Pairs of gilt ovaries were collected in warm 0.9% NaCl solution (Baxter, Old Toongabbie, NSW, Australia) from an abattoir. Cumulus–oocyte complexes (COCs) with diameters ranging from 3 to 6 mm were aspirated from follicles using an 18 G needle. The COCs were then washed three times in handling media (25 mM Hepes-TCM199; Gibco^®^, Thermo Fisher, Waltham, MA, USA) supplemented with 10% sow follicular fluid (SFF). COCs from individual ovary pairs were cultured in separate wells of a four-well plate containing 500 µL of pre-equilibrated in vitro maturation (IVM) media at 38.5 °C in a humidified incubator with 5% CO_2_ in air for 42 to 44 h. The IVM media comprised TCM199 media with 10 IU/mL of PMSG, 10 IU/mL of hCG, 0.10 µg/mL of EGF, 10% SFF, 0.80 mM Na-pyruvate, 0.61 mM L-glutamine, 0.88 M cysteamine, and 5 µg/mL of insulin.

For the collection of MII oocytes, expanded COCs were transferred into a 1.5 mL Eppendorf tube in 200 µL of handling media that also contained 5 µg/mL of hyaluronidase. After vortexing the COCs for 5 min, they were briefly spun down. The denuded oocytes were transferred to a dish and stripped further using a narrow glass pipette to completely remove all cumulus cells. Prior to microinjection, MII oocytes exhibiting a polar body were maintained in a microdroplet. At the time of microinjection, they were then transferred to a 20 µL droplet.

### 4.3. Isolation of Mitochondria from Oocytes

Mitochondrial fractions were purified from *Sus scrofa* oocytes, as described previously [5]. In essence, from 10 to 15 denuded oocytes which had not matured to the metaphase II stage in vitro were resuspended in 5 mL of mitochondrial isolation buffer consisting of 20 mM Hepes pH 7.6, 220 mM mannitol, 70 mM sucrose, 1 mM EDTA, and 2 mg/mL of BSA. Using a drill-fitted Potter-Elvehjem tissue grinder set (VWR International, West Chester, PA, USA) and on ice, the oocytes received 10 strokes to homogenise them. The homogenate was then centrifuged at 800× *g* for 10 min at 4 °C to remove debris. The supernatants were transferred into a new tube and centrifuged at 10,000× *g* for 20 min at 4 °C to pellet the mitochondria, which was then resuspended in 700 μL of mitochondrial isolation buffer and centrifuged for a further 20 min at 10,000× *g*. After removing the supernatant, the mitochondrial pellet was resuspended in 5 μL of mitochondrial isolation buffer in readiness for microinjection and DNA extraction.

### 4.4. Generation of Zygotes and Blastocysts

Oocytes were generated by intracytoplasmic sperm injection (ICSI) or ICSI along with the mitochondrial supplement, as previously described [5]. The mitochondrial supplement contained mtDNA from either sister oocytes (i.e., the same ovary) or third-party oocytes (i.e., another ovary pair). Once fertilised, and after 12 h of in vitro culture in an incubator, the resultant supplemented zygotes were transferred to a portable incubator set at 38.5 °C for transport for embryo transfer. For the generation of blastocysts, ICSI- and ICSI-supplemented zyogtes were cultured to the expanded blastocyst stage, as described in [30].

### 4.5. Synchronisation of Recipient Pigs

At 26 weeks of age, post-pubertal Large White × Landrace gilts were selected as recipients for embryo transfer. For each embryo transfer, two gilts underwent synchronisation to induce estrus. Synchronisation was through the oral administration of 0.22% altrenogest (Regumate; Merck Animal Health; Summit, NJ, USA) over a 16-day period. After the last altrenogest dose, 24 h later, 750 IU of eCG (Folligon; Intervet Australia, Bendigo, VIC, Australia) was administered followed by 750 IU of hCG (Chorulon; Intervet Australia) 104 h later to induce ovulation. Of the two gilts, the one exhibiting the strongest standing reflex and increased vulva swelling was selected as the recipient. Embryo transfer then took place 24 h after the administration of hCG, which was from 12 to 18 h before anticipated ovulation.

### 4.6. Surgery and Embryo Transfer

Following synchronisation, gilts were fasted overnight until surgery. They were administered thiopentone sodium intravenously at 15 mg/kg bodyweight (Thiobarb; Jurox Pty, Ltd., Rutherford, NSW, Australia) to induce anaesthesia, which was maintained using isoflurane (3.5% to 5%; Lyppard, Beverley, SA, Australia). The animal was then placed in a supine position on the surgical table and a 5 cm ventral midline incision was made between the two pairs of distal nipples. A uterine horn was isolated following the blunt dissection of the peritoneal cavity, which allowed the ovary and oviduct to be exteriorised. Zygotes were inserted into the oviduct by way of the infundibulum using a 11.4 cm Tom Cat catheter (Lyppard).

### 4.7. Post-Operative Care and Determination of Pregnancy

Once the recipient gilt had arisen, it was transferred to a single pen and administered antibiotic (150 mg/mL of amoxycillin trihydrate; Lyppard) and analgesic (50 mg/mL of flunixin meglumine; Lyppard) for up to 48 h post-surgery. On the 10th day after embryo transfer, 1000 IU of eCG was administered intramuscularly coupled with 1000 IU of hCG on the 13th day to generate accessory corpora lutea for the maintenance of pregnancy [66]. Pregnancy was assessed by real-time ultrasound between 28 and 45 days after embryo transfer; and confirmed by sighting amniotic vesicles.

### 4.8. Farrowing and Assessment at Birth

Throughout pregnancy, recipients received 2.5 kg/d of a commercial gestational diet. They were housed in pens up to 4 days prior to anticipated farrowing and transferred into a farrowing crate. Farrowing was supervised and induced using prostaglandin 2α (Lutalyse; Zoetis, NJ, USA) 116 days after embryo transfer if natural farrowing had not taken place.

### 4.9. RNA Extraction from Heart, RNAseq Library Construction, and Next-Generation Sequencing

Tissues were collected from the same location from each animal at the time of autopsy. The control pigs comprised two male pigs and one female pig generated through natural conception. They were sexually mature pigs of the same age as the mtDNA-supplemented pigs and tissues were collected from all animals at ~26 weeks of age. The tissues were then frozen in liquid N_2_ and stored in a −80 °C freezer until use. Circa 10 mg of tissue from control pigs (n = 3) and supplemented pigs (n = 6) was used to extract Total RNA using the RNeasy Mini Kit Qiagen Pty. Ltd., Clayton, VIC, Australia), as described by the manufacturer. The quality of the RNA was determined by a LabChipGX Nucleic Acid Analyzer (PerkinElmer Inc., Shelton, CT, USA) by assessing 28S, 18S, and 5S rRNA. RNAseq libraries were constructed and next-generation sequencing was performed by the Australian Genome Research Facility (Melbourne, VIC, Australia). Briefly, the depletion of rRNA in the RNAseq library was performed using the Ribo-zero stranded protocol (Illumina Inc., San Diego, CA, USA). Next-generation sequencing libraries were generated using 150 bp paired-end sequencing chemistry TruSeq SBS Kit v3 reagents (Illumina Inc.) and sequenced on the Illumina NovaSeq S1 platform. Cleaned sequence reads were then aligned against the pig genome (*Sus scrofa*—GCF_000003025.6. The STAR aligner v2.5.3a [67] was used to map reads to the genomic sequences. Raw gene counts or digital gene expression for use in edgeR were determined at the gene level using the featureCounts v1.5.3 utility of the subread package (http://subread.sourceforge.net/ (accessed on 1–26 March 2023)). The transcripts were assembled with the StringTie tool v2.1.4 (http://ccb.jhu.edu/software/stringtie/ (accessed on 1–26 March 2023)) utilising the aligned reads and reference annotation-based assembly option, which generates assembly for known and potentially novel transcripts. Differential gene expression was determined by edgeR (https://bioconductor.org/packages/release/bioc/html/edgeR.html (accessed on 1–26 March 2023)) using R v4.2.2.

### 4.10. DNA Extraction and Reduced Representation Bisulfite Sequencing

Total DNA was extracted from tissues using the QIAamp DNA Mini Kit (Qiagen Pty. Ltd., Hilden, Germany). RRBS libraries for 100 bp bar coded single-end runs were generated following the Nuggen’s Ovation^®^ RRBS Methyl-Seq System at the Australian Genome Research Facility and sequenced on an Illumina NovaSeq platform. Reads were assessed with FastQC v.0.11.8 and trimmed with Trim Galore v.0.6.7. Additional trimming was performed using Nugen’s diversity trimming script with default values. Mapping was carried out with Bismark v.0.22.3 [68] to the pig genome Sscrofa11.1_ensembl (release-110). Then, methylation calling was performed using Bismark. Differential methylation analysis was carried out using the Methylkit v.1.24.0 and edgeR v. 3.40.2 packages. Samples were imported into MethylKit session using a minimum read coverage of 8. The library sizes were corrected by the median of the total read counts for the methylated and un-methylated libraries. Differential methylation statistics were calculated using a logistic-regression-based model with over-dispersion correction and a Chi-square test between comparisons. In edgeR, the library sizes were corrected by the average of the total read counts for the methylated and un-methylated sites. A generalised linear model was then used to quantify the differential levels between the groups.

### 4.11. Gene Ontology, Functional Pathway Enrichment, and Gene Network Analysis

DEGs underwent GO analysis in edgeR. KEGG [69] pathway analysis, using over-representation analyses, was also conducted in edgeR. Reactome analysis was undertaken using reactome.org and the Analyse Gene List tool (accessed 26–28 August 2024) [70]. Analysis through Reactome was performed using official gene symbols, with non-human identifiers projected to human. For the GO and KEGG analyses, pathways with *p* < 0.05 were deemed to be over-represented in terms of the number of genes present in the pathway. For Reactome analysis, pathways with an FDR of < 0.05 were considered to be over-represented in the context of the number of genes present in the pathway. For both KEGG and Reactome, over-representation analyses were run solely for the upregulated genes or downregulated genes, as these allow for the greatest statistical power for discovering biologically significant pathways [27]. However, all pathways (upregulated and downregulated) were also combined in one table (Supplementary Tables S8C and S9C) for the Reactome analysis to provide an overall overview of the total number of significant pathways affected and their distribution across tissues.

### 4.12. mtDNA Amplification

Four separate reactions were performed to amplify the whole mitochondrial genome of oocytes and tissues. Total DNA was extracted from oocytes using the QIAamp DNA Micro Kit (Qiagen Pty. Ltd.). Total DNA was extracted from tissues using the QIAamp DNA Mini Kit (Qiagen Pty. Ltd.). For each oocyte, 4 µL of total DNA was used per reaction. For tissues, 50 ng of total DNA was used per reaction. Reactions were performed using the following components: 5 µL of 10× High Fidelity buffer, 2 µL of 50 mM MgSO_4_, 1 µL 10 mM dNTP mix (Meridian Bioscience, Millennium Science Australia Pty. Ltd., Mulgrave, VIC, Australia), 1 µL of each primer at 10 µM (500 nM each), as described in [28], 35.8 µL of autoclaved Milli-Q water, and 0.2 µL of High-Fidelity *Taq* polymerase (Thermo Fisher Scientific Australia Pty. Ltd., Scoresby, VIC, Australia). PCR amplification was performed in an MJ Research PTC-200 dual-block thermal cycler according to the cycling conditions described in [28].

Following amplification, 15 µL of each sample were loaded into a 1% agarose (Meridian Bioscience) gel with 0.5× TAE running buffer and SYBR-Safe (Thermo Fisher Scientific, Waltham, MA, USA) and run at 100 V for ~1 h. The presence of the amplified product was confirmed by a GelDoc EX imager set at 100 ms exposure and compared to 10 KB Plus ladder (Thermo Fisher Scientific). The remaining 35 µL of PCR product was purified using the ISOLATE II PCR and Gel Kit (Meridian Bioscience), according to the manufacturer’s instructions, and eluted into 15 µL of autoclaved Milli-Q water. The DNA concentration was determined by Qubit DNA HS 1X (Thermo Fisher Scientific) using a Qubit 2.0 fluorometer, according to the manufacturer’s instructions. Each fragment was then combined to produce 50 ng of total product, with 3 ng per kb of template (i.e., 21.78 ng of fragment 1, 9.39 ng of fragment 2, 9.69 ng of fragment 3, and 10.98 ng of fragment 4).

### 4.13. mtDNA Next Generation Sequencing

Library construction and sequencing were carried out by the Australian Genome Research Facility. Initial quality control was performed using 1% E-Gel (Thermo Fisher Scientific Australia Pty. Ltd.) to confirm the presence of amplicons, in addition to the confirmation of DNA concentration by the QuantiFluor^®^ dsDNA system (Promega Corporation, Alexandria, NSW, Australia). After initial quality control, 9 µL was used for library preparation using Prep-M library preparation, with 12 amplification cycles (Nextera DNA Flex, Illumina Inc.) to generate mtDNA amplicon libraries. These were sequenced on the Illumina NovaSeq X platform using the NovaSeq X 10B lane (300 cycles, 150 bp paired-end reads).

### 4.14. mtDNA Mapping

Sequences were aligned in CLC Genomics Workbench Version 23.0.5 (Qiagen Pty. Ltd.) to AJ002189.1, a porcine reference pig mitochondrial genome [71]. The 5′ and 3′ ends of the reads were trimmed by one nucleotide. Reads of <15 bp in length were excluded. Only reads that surpassed a Phred quality score of 15 were included in mapping. The mapping parameters were a match score of 1; a mismatch cost of 2; an insertion/deletion cost of 3; and a similarity fraction of 0.80, which led to 98% of the reads aligning to the reference genome. Duplicate reads were then excluded. For each sample, a consensus sequence (reference genome) was generated and the trimmed reads were remapped against this sequence, including the removal of duplicate reads. Consensus sequences were aligned using the Create Alignment tool (CLC Genomics Workbench, Qiagen PTY Ltd.) and using a gap cost of 10 and gap extension cost of 1, which were then used to generate phylogenetic trees constructed after the import of alignments into the Maximum Likelihood Phylogeny tool (CLC Genomics Workbench). Trees were generated using the Neighbour Joining method and the General Time Reversible nucleotide substitution model with the bootstrapping of 1000 replicates.

### 4.15. Analysis of Heteroplasmic Load

Heteroplasmic load was determined from the reads mapped to each individual’s consensus sequence using the Low-Frequency Variant Detection module (CLC Genomic Workbench) with the following parameters: the exclusion of all duplicate reads; the presence of all variants on forward and reverse reads; a minimum of 50 reads for each variant; and a minimum threshold of 2.0% for the calling of each variant.

## Figures and Tables

**Table 1:** Each pig and mitochondrial preparation underwent next-generation mtDNA sequencing. In this case, the sequencing was used to construct a whole mtDNA genome consensus sequence for each pig that was then compared to the consensus sequence of the respective MP sample to demonstrate that either autologous or heterologous mtDNA supplementation had taken place, as shown in Figure 1 and Figure 2, respectively. The mtDNA sequence was also used to assign the pigs to one of the commercially known pig mtDNA haplotypes. * Pigs 3.2 and 3.5 are very closely aligned to haplotype KT279759 and likely belong to a sub-clade of this haplotype.

Pig ID	Mitochondrial Preparation	Autologous or Heterologous	Assigned mtDNA Haplotype
Pig 3.1	MP3	Heterologous	KT279758
Pig 3.2	MP3	Heterologous	KT279759 *
Pig 3.3	MP3	Heterologous	KT261430
Pig 3.4	MP3	Autologous	KT261429
Pig 3.5	MP3	Heterologous	KT279759 *
12.1	MP12	Autologous	KT279760
12.2	MP9	Autologous	KT261430
12.3	MP13	Autologous	KT279758
12.4	MP12	Autologous	KT279760
17.1	MP14	Autologous	Unassigned
17.2	MP14	Autologous	Unassigned
17.3	MP14	Autologous	Unassigned

**Table 2:** Differentially expressed genes (FDR < 0.05) common to two (italicised) and three tissues (bold) for all offspring against controls. Red represents significantly upregulated and blue significantly downregulated, as indicated in Figure 3. Ranking of significance for gene expression is shown in Supplementary Table S4.

Brain	Heart	Liver	Function
**LOC110260659**	**LOC110260659**	**LOC110260659**	Translationally controlled tumour protein pseudogene
**LOC100518848**	**LOC100518848**	**LOC100518848**	40S ribosomal protein S21
*LOC110258138*		*LOC110258138*	non-coding RNA
*SGCA*		*SGCA*	Sarcoglycan Alpha (stability of muscle fibre membranes)
	*LOC106505031*	*LOC106505031*	non-coding RNA
	*PDZD9*	*PDZD9*	PDZ Domain Containing 9 (Mitochondrial Complex V Deficiency, Nuclear Type 3 and Meckel Syndrome, Type 4)

**Table 3:** Differentially expressed genes common to two (underlined) and three (bold) tissues in the comparison between heterologous and autologous offspring (FDR < 0.05). Red represents significantly upregulated and blue significantly downregulated gene expression, as indicated in Figure 5. Ranking of significance for gene expression is shown in Supplementary Table S6.

Brain	Heart	Liver	
* ACSM5 *	* ACSM5 *		Acyl-Coenzyme A Synthetase ACSM5, Mitochondrial
**LOC102164558**	**LOC102164558**	**LOC102164558**	ncRNA
**LOC100525311**	**LOC100525311**	**LOC100525311**	Golgi phosphoprotein 3 (GOLPH3)
* IGF2BP3 *		* IGF2BP3 *	Insulin Like Growth Factor 2 MRNA Binding Protein 3
**LOC100152036**	**LOC100152036**	**LOC100152036**	SLAM family member 9-like (SLAMF9)
* KIRREL *	* KIRREL *		Kirre Like Nephrin Family Adhesion Molecule 1
**LOC106508088**	**LOC106508088**		ncRNA
* TLE4 *		* TLE4 *	TLE Family Member 4, Transcriptional Corepressor
	* NTRK2 *	* NTRK2 *	Neurotrophic Receptor Tyrosine Kinase 2
	* TRIM7 *	* TRIM7 *	Tripartite Motif Containing 7
	* NEXN *	* NEXN *	Nexilin F-Actin Binding Protein
	LOC106509396	LOC106509396	Uncharacterised, Protein coding
	* COG7 *	* COG7 *	Component Of Oligomeric Golgi Complex 7
	LOC110261018	LOC110261018	Uncharacterised, Protein coding
	* EMILIN3 *	* EMILIN3 *	Elastin Microfibril Interface-Located Protein 3
	LOC106506003	LOC106506003	ncRNA
	* RRBP1 *	* RRBP1 *	Ribosome Binding Protein 1
	LOC102163231	LOC102163231	ncRNA
	* FLRT3 *	* FLRT3 *	Fibronectin Leucine Rich Transmembrane Protein 3
	LOC110258845	LOC110258845	ncRNA
	LOC106506130	LOC106506130	ncRNA
	* SPAG16 *	* SPAG16 *	Sperm Associated Antigen 16

**Table 4:** The top 10 upregulated and downregulated pathways as determined by Reactome for all offspring when compared against controls at FDR < 0.05 (range from 0.001 to 0.024).

Downregulated		
Brain	Heart	Liver
Inhibition of TSC complex formation by PKB	Resolution of AP sites via the single-nucleotide replacement pathway	Regulation of cytoskeletal remodelling and cell spreading by IPP complex components
APC/C:Cdh1-mediated degradation of Cdc20 and other APC/C:Cdh1 targeted proteins in late mitosis/early G1	Synthesis of PS	The AIM2 inflammasome
Defective C1GALT1C1 causes TNPS	Hydrolysis of LPC	The IPAF inflammasome
VxPx cargo-targeting to cilium	Glycerophospholipid biosynthesis	MET promotes cell motility
TNFR2 non-canonical NF-kB pathway	Abasic sugar-phosphate removal via the single-nucleotide replacement pathway	Collagen biosynthesis and modifying enzymes
TNFs bind their physiological receptors	SUMOylation of DNA replication proteins	Phospholipase C-mediated cascade; FGFR3
tRNA modification in the nucleus and cytosol	NPAS4 regulates expression of target genes	PLC-gamma1 signalling
Ethanol oxidation	Host Interactions of HIV factors	MET activates PTK2 signalling
Defective F8 binding to von Willebrand factor	Regulation of signalling by NODAL	Alternative complement activation
**Upregulated**		
**Brain**	**Heart**	**Liver**
Elevation of cytosolic Ca2+ levels	Presynaptic depolarization and calcium channel opening	Vif-mediated degradation of APOBEC3G
Autodegradation of Cdh1 by Cdh1:APC/C	Organic cation transport	Regulation of apoptosis
APC/C:Cdc20-mediated degradation of Securin	Dual incision in GG-NER	Regulation of activated PAK-2p34 by proteasome-mediated degradation
Transport and synthesis of PAPS	Organic cation/anion/zwitterion transport	Branched-chain amino acid catabolism
APC/C:Cdc20-mediated degradation of mitotic proteins	Choline catabolism	Abnormal conversion of 2-oxoglutarate to 2-hydroxyglutarate
Activation of APC/C and APC/C:Cdc20-mediated degradation of mitotic proteins	Ciprofloxacin ADME	Ubiquitin-mediated degradation of phosphorylated Cdc25A
APC:Cdc20-mediated degradation of cell cycle proteins prior to satisfaction of the cell cycle checkpoint	Transcription-coupled nucleotide excision repair (TC-NER)	p53-independent DNA damage response
Downregulation of TGF-beta receptor signalling	Defective MMAA causes MMA, cblA type	p53-independent G1/S DNA damage checkpoint
Metabolism of ingested H2SeO4 and H2SeO3 into H2Se	Defective MUT causes MMAM	Cross-presentation of soluble exogenous antigens (endosomes)
DSCAM interactions	Diseases of mitochondrial beta oxidation	Hh mutants abrogate ligand secretion

**Table 5:** The top 10 upregulated and downregulated pathways as determined by Reactome for the comparison between heterologous and autologous offspring (FDR = 0.001 for all pathways).

Downregulated		
Brain	Heart	Liver
Methionine salvage pathway	Phase II—conjugation of compounds	Opioid signalling
Mitochondrial iron–sulfur cluster biogenesis	Cytosolic sulfonation of small molecules	Calmodulin-induced events
Amino acid conjugation	Transfer of LPS from LBP carrier to CD14	Cam-PDE 1 activation
L13a-mediated translational silencing of ceruloplasmin expression	Nucleotide-binding domain, leucine-rich repeat-containing receptor (NLR) signalling pathways	Ca-dependent events
Conjugation of carboxylic acids	DDX58/IFIH1-mediated induction of interferon-alpha/beta	CaM pathway
Conjugation of salicylate with glycine	Regulation of pyruvate–dehydrogenase (PDH) complex	G-protein mediated events
Signalling by FGFR2 amplification mutants	Biological oxidations	PLC beta mediated events
Activated point mutants of FGFR2	Defective GGT1 causes GLUTH	Neurotransmitter receptors and postsynaptic signal transmission
MicroRNA (miRNA) biogenesis	RHOG GTPase cycle	Cross-presentation of particulate exogenous antigens (phagosomes)
Regulation of gene expression in endocrine-committed (NEUROG3+) progenitor cells	Defective GGT1 in aflatoxin detoxification causes GLUTH	SHC1 events in ERBB2 signalling
**Upregulated**		
**Brain**	**Heart**	**Liver**
Cam-PDE 1 activation	Collagen degradation	ER-Phagosome pathway
G-protein-mediated events	Degradation of the extracellular matrix	Antigen processing cross presentation
PLC beta-mediated events	Collagen formation	Signalling by NOTCH
Presynaptic depolarization and calcium channel opening	Elastic fibre formation	Disease
Nuclear signalling by ERBB4	Activation of matrix metalloproteinases	Complement cascade
Tandem of pore domain in a weak inwardly rectifying K+ channels (TWIK)	Sulphide oxidation to sulphate	Activation of C3 and C5
Sperm motility and taxes	Glycosaminoglycan metabolism	Pre-NOTCH processing in Golgi
Regulation of signalling by NODAL	Collagen biosynthesis and modifying enzymes	Pre-NOTCH expression and processing
ARMS-mediated activation	Complement cascade	Cholesterol biosynthesis
FGFR1c and Klotho ligand binding and activation	NGF processing	Downstream TCR signalling

**Table 6:** Overlapping commonly differentially expressed genes and DNA-methylated regions for all offspring compared with controls and heterologous compared with autologous offspring. Blue font indicates downregulation and red font highlights upregulation in gene expression. Underlined indicates the presence of a gene in two tissues. Genes are listed with the most significantly different displayed first.

All Offspring			Heterologous v Autologous		
Brain	Heart	Liver	Brain	Heart	Liver
*ADRM1*	*CHRNA4*	*PTK6*	* ACSM5 *	* ACSM5 *	*KCND3*
*RPS21*	*MYT1*	*KIF1A*	*SLAMF9*	*TOP1MT*	*PARVB*
		*ABCA13*		*DGKI*	*DLGAP2*
		*NOS2*		*PRKAR1B*	*SGF29*
		*DSCAM*		*MYT1L*	*SLC26A2*
		*NAV3*		*CDS1*	*NAV2*
		*SDK1*		*MLYCD*	*TTBK1*
				*HS3ST2*	*CLDN10*
				*MICU2*	*SYT13*
					*CRELD2*
					*PC*
					*ATP8A2*
					*CACNB2*
					*LRRC8D*
					*SMURF1*
					*PPFIBP2*
					*RGS17*
					*MBOAT2*
					*CFAP46*

**Table 7:** Common overlapping DMRs identified in expanded blastocysts and methylated sites in all three and any two tissues for all offspring compared with controls and heterologous compared with autologous offspring. Bold indicates the presence of a gene in two comparisons.

Three Tissues		Two Tissues	
All Offspring	Heterologous vs. Autologous	All Offspring	Heterologous vs. Autologous
*TAFA5*	*RALGDS*	*SHANK2*	*ARID1B*
*DST*	*JAKMIP2*	*FSTL4*	*CDK5RAP2*
***PITRM1***	*ARHGEF37*	*GSE1*	*PPP6R3*
*COG3*	*PPARGC1B*	*SACS*	*DPP3*
*BNIP3*	*PDE6A*	*ABCA13*	*MACROH2A1*
*COL4A4*	*HMGXB3*	*TCERG1L*	*HTR4*
*STMN3*	*CSF1R*	*NDUFA10*	*ABLIM3*
***PTPRN2***	*CDX1*	*CDH4*	*SUN1*
	*TCOF1*	*TPD52L2*	*SDK1*
	*SYNPO*	*SHROOM2*	*AUTS2*
	*FOXK1*	*DCDC2C*	*IQCK*
	*RADIL*	*NAV3*	*TXNDC11*
	*TRRAP*	*SORCS2*	*CIITA*
	*GTF3C1*	*ADARB2*	*TBC1D8*
	*SNX29*		*ST18*
	*CLEC16A*		*TBC1D22A*
	*DNMT3A*		*TTLL1*
	*OSR1*		*USP10*
	*ZNHIT6*		*PLCG2*
	*CBFA2T3*		*CDYL2*
	*PEPD*		*CAMTA1*
	*SORCS2*		*LUZP1*
	*LEF1*		*RGS12*
	***PITRM1***		*TBC1D1*
	*PFKP*		*PPFIBP2*
	*ADARB2*		*NMNAT2*
	*DIP2C*		*LNX2*
	*SACS*		*SLC7A1*
	*MIPEP*		*KCNJ2*
	*ATP8A2*		*MSI2*
	*DNAH17*		*SLC5A10*
	*SEPTIN9*		*MAP2K3*
	*MGAT5B*		*HSPA12A*
	*SPECC1*		*DLGAP2*
	*MTUS2*		*INPP5D*
	*USP36*		*SLC12A7*
	*MGMT*		*PCBP3*
	*AGAP1*		*PDE1C*
	*NDUFA10*		*COG3*
	*CDH4*		*KSR1*
	***PTPRN2***		*C10orf90*
	*DPP6*		*HDAC4*
	*DGKI*		*NDUFS6*
			*PHACTR3*
			*SND1*
			*AFAP1L1*
			*SMURF1*
			*EPS8L3*
			*LRRC8D*
			*TAFA5*
			*CRISPLD2*
			*WWOX*
			*STX18*

## Data Availability

The data sets supporting the conclusions of this article are available in the NCBI Sequence Read Archive (https://www.ncbi.nlm.nih.gov/sra) under BioProjects ID PRJNA823749; NCBI BioProject: PRJNA749323; BioProject: PRJNA1214402.

## Supplementary Materials

The following supporting information can be downloaded at: https://www.mdpi.com/article/10.3390/ijms26062746/s1.
